# spatialLIBD: an R/Bioconductor package to visualize spatially-resolved transcriptomics data

**DOI:** 10.1186/s12864-022-08601-w

**Published:** 2022-06-10

**Authors:** Brenda Pardo, Abby Spangler, Lukas M. Weber, Stephanie C. Page, Stephanie C. Hicks, Andrew E. Jaffe, Keri Martinowich, Kristen R. Maynard, Leonardo Collado-Torres

**Affiliations:** 1 Licenciatura de Ciencias Genómicas, Escuela Nacional de Estudios Superiores Unidad Juriquilla, Universidad Nacional Autónoma de México, Querétaro, 76230 Mexico; 2 Lieber Institute for Brain Development, Johns Hopkins Medical Campus, Baltimore, 21205 Maryland USA; 3 Department of Biostatistics, Johns Hopkins Bloomberg School of Public Health, Baltimore, 21205 Maryland USA

**Keywords:** Spatially-resolved transcriptomics, Interactive visualization, 10x Genomics Visium

## Abstract

**Background:**

Spatially-resolved transcriptomics has now enabled the quantification of high-throughput and transcriptome-wide gene expression in intact tissue while also retaining the spatial coordinates. Incorporating the precise spatial mapping of gene activity advances our understanding of intact tissue-specific biological processes. In order to interpret these novel spatial data types, interactive visualization tools are necessary.

**Results:**

We describe *spatialLIBD*, an R/Bioconductor package to interactively explore spatially-resolved transcriptomics data generated with the 10x Genomics Visium platform. The package contains functions to interactively access, visualize, and inspect the observed spatial gene expression data and data-driven clusters identified with supervised or unsupervised analyses, either on the user’s computer or through a web application.

**Conclusions:**

*spatialLIBD* is available at https://bioconductor.org/packages/spatialLIBD. It is fully compatible with *SpatialExperiment* and the Bioconductor ecosystem. Its functionality facilitates analyzing and interactively exploring spatially-resolved data from the Visium platform.

**Supplementary Information:**

The online version contains supplementary material available at (10.1186/s12864-022-08601-w).

## Background

The rise of spatially-resolved transcriptomics technologies [1, 2] is providing new opportunities to answer questions about the structure and function of complex intact tissues [3–5]. However, this exciting opportunity requires the development of new computational methods and interactive software. These tools have to accommodate the analysis of transcriptomic data with spatial coordinates and high resolution images into previously developed methods for single cell RNA-sequencing (scRNA-seq) analysis, which lacked these components.

Currently there are limited options for interactive exploration of spatially-resolved transcriptomics data. The 10x Genomics Loupe Browser [6] utilizes a file created by the Space Ranger data pre-processing pipeline [6] and performs marker gene analysis, as well as unsupervised graph-based and *k*-means clustering. Loupe visualizes both the spatially-resolved transcriptomics data along with the high resolution imaging data to export gene expression maps. An alternative to Loupe is the Giotto pipeline [7], which contains both a data analysis and data visualization modules. The data analysis module has many functions ranging from pre-processing to more advanced analysis such as characterizing cell-cell interactions. The visualization module contains an interactive workspace for exploring multiple layers of information. However, Loupe and Giotto currently do not support visualizing more than one tissue section at a time, which is useful for comparing replicates and annotating observed spots on the Visium array across samples. Seurat [8] provides guidelines for processing and visualizing spatially-resolved transcriptomics data at https://satijalab.org/seurat/articles/spatial_vignette.html. Seurat [8] does not however provide interactive visualizations or the ability to create an interactive website. Furthermore, while unsupervised clustering methods are widely developed [9, 10], manual annotation of spots using known marker genes is important, as well as cross-sample dimension reduction techniques such as UMAP and t-SNE.

## Implementation

*spatialLIBD* is an R/Bioconductor [11] package that provides functions to import output from Space Ranger [6] including clustering (k-means, graph-based) and dimension reduction results (PCA, UMAP, t-SNE), interactively visualize and explore spatially-resolved transcriptomics data, and provides functionality to manually annotate spots. It was developed initially for visualizing multiple Visium tissue sections from the human brain [4], yet it is flexible enough to be used with other Visium data from different tissue types (Supplementary File 1, Fig. 1A).

**Fig. 1:**
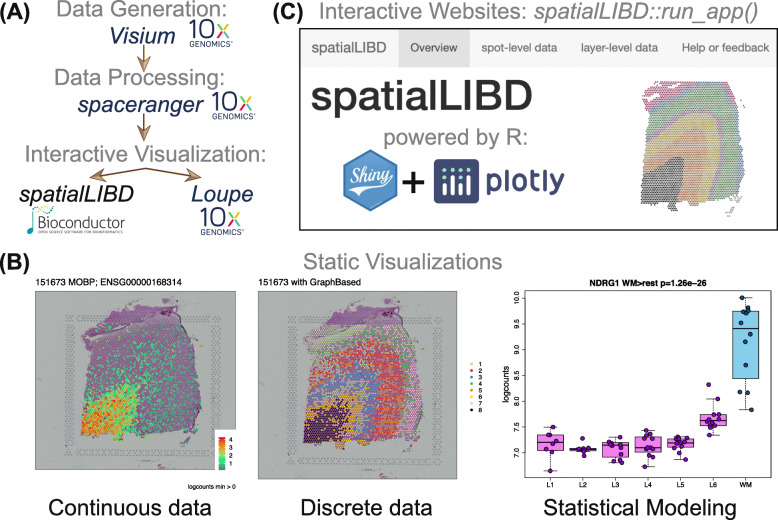
Overview of *spatialLIBD*: **A** Using spatially-resolved transcriptomics data generated with the *Visium* platform and processed with *spaceranger*, both from 10x Genomics, *spatialLIBD* leverages the R/Bioconductor ecosystem and presents an alternative for interactive visualization to *Loupe Browser*. **B***spatialLIBD* supports static data visualizations of both continuous and categorical measurements along with the histology images, as well as visualizing results from downstream analyses. **C***spatialLIBD* supports interactive websites for exploring the data powered by *shiny* and *plotly*

The primary functions in *spatialLIBD* allow users (i) to visualize the spot-level spatial gene expression data or any other continuous variable, and data-driven clusters or any categorical variable, with the histology image in the background (Fig. 1B), and (ii) to inspect the data interactively, either on the user’s computer or through web hosting services such as *shinyapps.io* [12] (Fig. 1C). In addition, *spatialLIBD* enables users to visualize multiple samples at the same time, to export all static visualizations as PDF files or all interactive visualizations as PNG files, as well as all result tables as CSV files. Additional file 1 shows how to read in data processed with Space Ranger [6] into R, make plots or create an interactive website with *spatialLIBD*.

Additional file 2 illustrates how *spatialLIBD* can be used to visualize clustering results derived from multiple samples as well as other continuous covariates, using a human brain dataset with twelve samples [4]. In particular, clusters derived from all samples facilitate their interpretation since cluster numbers (and thus their meaning) will be the same across all samples. The companion interactive website for this dataset is available at http://spatial.libd.org/spatialLIBD. Additional file 1 shows the steps necessary to make your own interactive website, such as https://libd.shinyapps.io/spatialLIBD_Human_Lymph_Node_10x/.

**Fig. 2:**
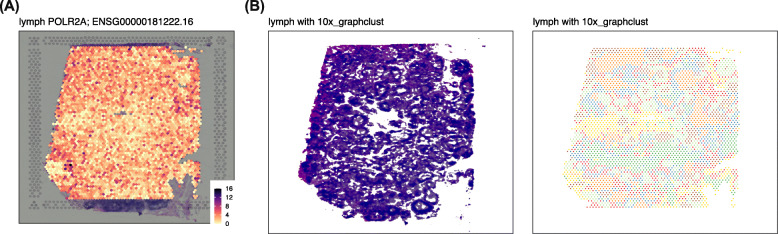
Enhanced visualization *spatialLIBD* features: Using the example *spatialLIBD* web application from https://libd.shinyapps.io/spatialLIBD_Human_Lymph_Node_10x/ we illustrate some recent enhancements. The data is from the human lymph node example dataset by 10x Genomics. **A** Expression of the *POLR2A* gene with a point size of 2.25 and using the reversed *magma* colorblind-friendly continuous scale: low values are in white. There are nine color scales options and each can be reversed. The transparency of the spots can be controlled as well. **B** A side-by-side view of the histology and the graph-based clusters produced by Space Ranger. The original *lowres* image from Space Ranger has been edited with *spatialLIBD*: *enhance*, *normalize*, *equalize*, *transparent color* pink, and *transparent fuzz* 40

*spatialLIBD* has evolved over time and now provides features that further facilitate the visual inspection of the results. These include nine color scales, including colorblind-friendly scales as well as the option to reverse the color scale gradient order (Fig. 2A). The transparency of the spots can be controlled as well. Multiple images are supported and can be edited on the web application to increase brightness or perform other image manipulations that can reveal hidden patterns to the eye (Fig. 2B). Side by side plots of the images and the clusters or gene expression data can also be generated.

*spatialLIBD* is designed to work with *SpatialExperiment* R/Bioconductor objects [13]. It uses *shiny* [14] and *plotly* [15] for the interactive website, *ggplot2* [16] and *cowplot* [17] for the static images. Interactive image editing is powered by *magick* [18]. Colorblind-friendly colors are provided by *viridis* [19].

## Discussion

*spatialLIBD* benefits from the open-source Bioconductor ecosystem [11] and relies on the infrastructure provided by *SpatialExperiment* [13]. It provides user-friendly functionality for visualizing continuous and categorical measurements along with the tissue images from spatially-resolved transcriptomics data from projects involving one or more samples measured on the 10x Genomics Visium platform. Since *spatialLIBD* is compatible with *SpatialExperiment*, *spatialLIBD* could be used for other spatially-resolved transcriptomic platforms as they become better supported by *SpatialExperiment*.

*spatialLIBD* provides a simple interactive website, which can be used for sharing data on the web, as well as manually annotating spots. *spatialLIBD* has limitations (Additional file 1) that are inherent to the methods used to implement it, such as (i) the memory per user required by a server for hosting the web application, (ii) response speeds for the interactive views due to the number of spots, (iii) the resolution of the images displayed limiting the usefulness to magnify specific spots, and (iv) customization of the web application by the end user. Despite these limitations, open-source software solutions like *spatialLIBD* enable faster data exploration and insights in spatially-resolved transcriptomics research projects [4] and can serve as a testing ground for ideas.

While some visualization software alternatives do not provide visualizations across multiple samples, static visualizations of multiple samples can be combined using R packages such as *cowplot* or *patchwork* or interactive versions using *plotly*, which is how *spatialLIBD* was implemented. As spatially-resolved transcriptomics becomes more widely available [20], new methods are being implemented in other languages, such as Python. One such example is *squidpy* [21], which is currently limited to one Visium sample as noted at https://github.com/theislab/squidpy/issues/417. *squidpy* does provide exciting and powerful features, and bridges the R and Python worlds.

## Conclusion

*spatialLIBD* is fully integrated with the R/Bioconductor open source ecosystem and is available at https://bioconductor.org/packages/spatialLIBD. It provides user-friendly functionality for importing results from the 10x Genomics Space Ranger pipeline for processing spatially-resolved transcriptomics data from Visium. *spatialLIBD* provides different options for visualizing and interactively exploring the data from multiple Visium capture areas, and can create websites that can be self-hosted such as http://spatial.libd.org/spatialLIBD/ or hosted at *shinyapps.io* such as the example website https://libd.shinyapps.io/spatialLIBD_Human_Lymph_Node_10x/.

## Availability and requirements

**Project name:***spatialLIBD*: an R/Bioconductor package to visualize spatially-resolved transcriptomics data

**Project home page:** https://bioconductor.org/packages/spatialLIBD

**Operating system(s):** Platform independent

**Programming language:** R

**Other requirements:** Bioconductor version 3.13 or newer to access the latest features

**License:** Artistic-2.0

**Any restrictions to use by non-academics:** none

## Supplementary Information

**Additional file 1** Using *spatialLIBD* with 10x Genomics public datasets. Vignette document showing a detailed tutorial on how to use public 10x Genomics Visium data and visualize it using *spatialLIBD*.

**Additional file 2** Visualizing multiple samples with *spatialLIBD*. R code and resulting PDF files for visualizing across all 12 human brain samples from Maynard *et al* [4] (a) clusters derived from a shared-nearest neighbor (k = 50) that was computed using the genomic data from all samples, (b) the percent of mitochondrial expression which has a spatial distribution that matches the cortical layers, and (c) the expression of *MOBP* which is higher in the white matter than other layers [4].

## Data Availability

10x Genomics provides public data examples for their Visium platform, such as the lymph node example used here https://support.10xgenomics.com/spatial-gene-expression/datasets/1.1.0/V1_Human_Lymph_Node.

## Abbreviations

scRNA-seq: single cell RNA sequencing
