# Supplementary material for: spatialLIBD: an R/Bioconductor package to visualize spatially-resolved transcriptomics data
Source: BMC Genomics. 2022 Jun 10;23:434. doi: 10.1186/s12864-022-08601-w (PMC9188087; doi:10.1186/s12864-022-08601-w)
Supplement: Supplementary file 2 — Additional file 2 Visualizing multiple samples with spatialLIBD. R code and resulting PDF files for visualizing across all 12 human brain samples from Maynard et al [4] (a) clusters derived from a shared-nearest neighbor (k = 50) that was computed using the genomic data from all samples, (b) the percent of mitochondrial expression which has a spatial distribution that matches the cortical layers, and (c) the expression of MOBP which is higher in the white matter than other layers [4]. [file 12864_2022_8601_MOESM2_ESM.pdf]

```
library("spatialLIBD")

## Download the Maynard et al, Nat Neurosci, 2021 data
spe <- fetch_data("spe")

## Visualize the result from a shared-nearest neighbor (k = 50) graph
## cut at 7 clusters (k7), hence SNN_k50_k7. This graph was constructed
## across all samples, such that cluster 1 on sample 1 has the same meaning
## as cluster 1 on samples 2, 3, ... n.
vis_grid_clus(
    spe = spe,
    clustervar = "SNN_k50_k7",
    pdf_file = "grid_SNN_k50_k7.pdf",
    colors = setNames(Polychrome::palette36.colors(7), seq_len(7)),
    point_size = 2,
    alpha = 0.75
)

## Visualize the chrM expression ratio across all samples with the
## "magma" color palette in the reverse direction (-1) such that darker colors
## represent higher values
vis_grid_gene(
    spe = spe,
    geneid = "expr_chrM_ratio",
    pdf_file = "grid_expr_chrM_ratio.pdf",
    minCount = -1,
    cont_colors = viridisLite::viridis(21, direction = -1, option = "magma"),
    point_size = 2
)

## Visualize the normalized expression values (logcounts) for the gene
## MOBP which has a higher expression in the white matter. Here we use the
## "plasma" color palette with the regular direction (+1) such that
## higher values are represented with brighter colors.
vis_grid_gene(
    spe = spe,
    geneid = rowData(spe)$gene_search[rowData(spe)$gene_name == "MOBP"],
    pdf_file = "grid_MOBP.pdf",
    minCount = -1,
    cont_colors = viridisLite::viridis(21, direction = 1, option = "plasma"),
    point_size = 2
)

## For reproducibility
packageVersion("spatialLIBD")
# [1] â€˜1.7.13â€™
```

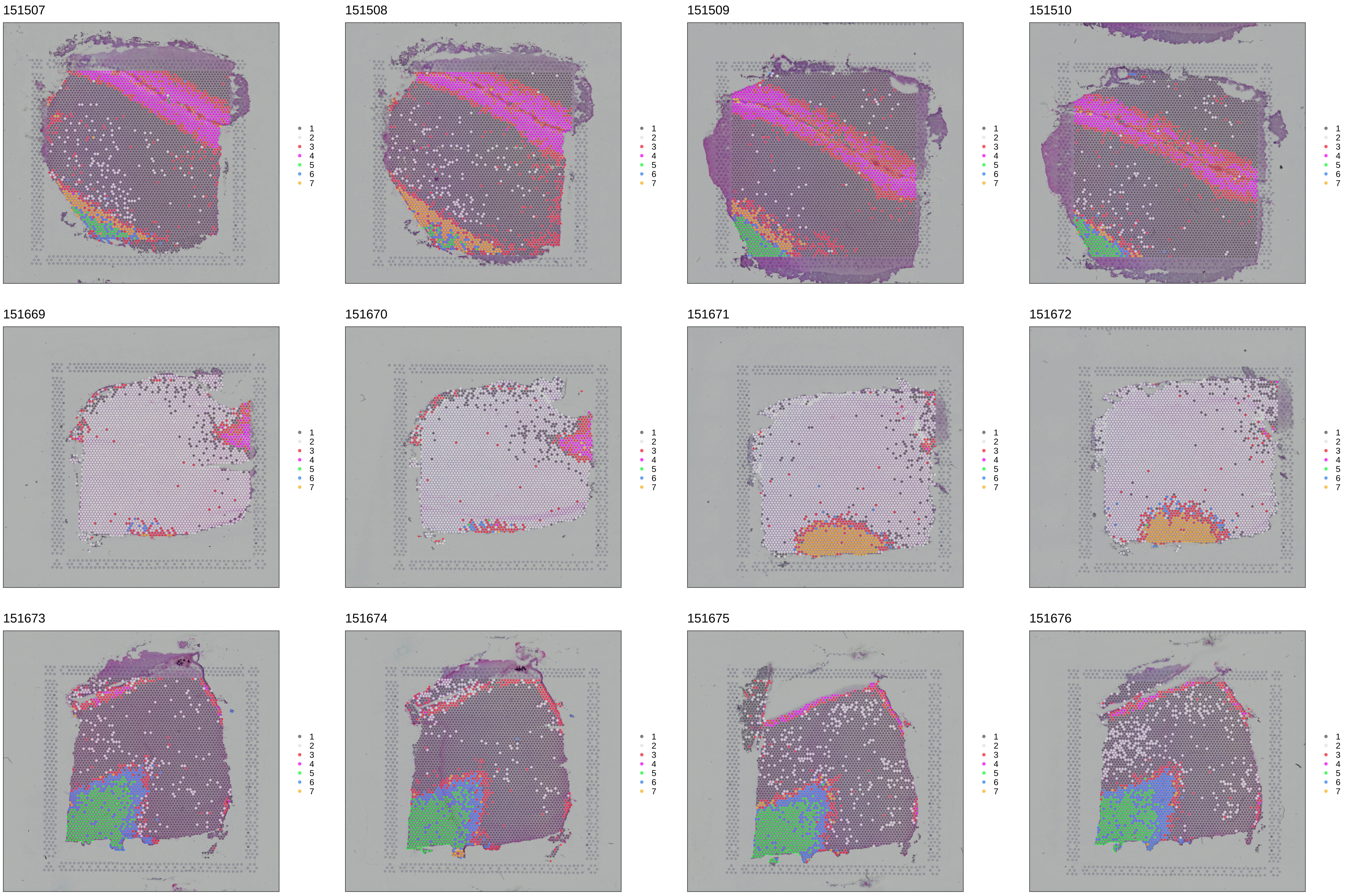
151507
1
2
3
4
5
6
7
151508
1
2
3
4
5
6
7
151509
1
2
3
4
5
6
7
151510
1
2
3
4
5
6
7
151669
1
2
3
4
5
6
7
151670
1
2
3
4
5
6
7
151671
1
2
3
4
5
6
7
151672
1
2
3
4
5
6
7
151673
1
2
3
4
5
6
7
151674
1
2
3
4
5
6
7
151675
1
2
3
4
5
6
7
151676
1
2
3
4
5
6
7

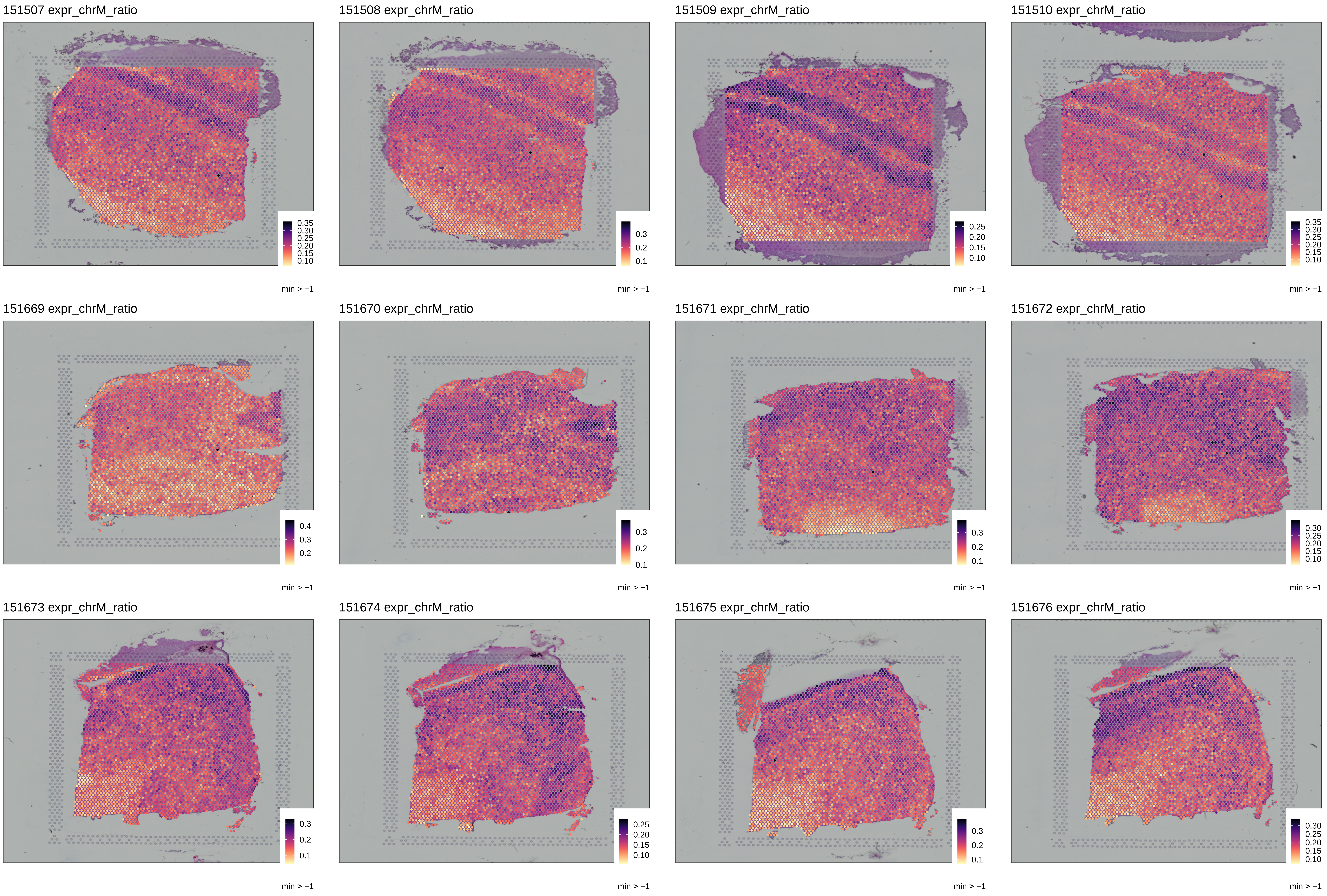
151507 expr_chrM_ratio
0.35
0.30
0.25
0.20
0.15
0.10
min > −1
151508 expr_chrM_ratio
0.3
0.2
0.1
min > −1
151509 expr_chrM_ratio
0.25
0.20
0.15
0.10
min > −1
151510 expr_chrM_ratio
0.35
0.30
0.25
0.20
0.15
0.10
min > −1
151669 expr_chrM_ratio
0.4
0.3
0.2
min > −1
151670 expr_chrM_ratio
0.3
0.2
0.1
min > −1
151671 expr_chrM_ratio
0.3
0.2
0.1
min > −1
151672 expr_chrM_ratio
0.30
0.25
0.20
0.15
0.10
min > −1
151673 expr_chrM_ratio
0.3
0.2
0.1
min > −1
151674 expr_chrM_ratio
0.25
0.20
0.15
0.10
min > −1
151675 expr_chrM_ratio
0.3
0.2
0.1
min > −1
151676 expr_chrM_ratio
0.30
0.25
0.20
0.15
0.10
min > −1

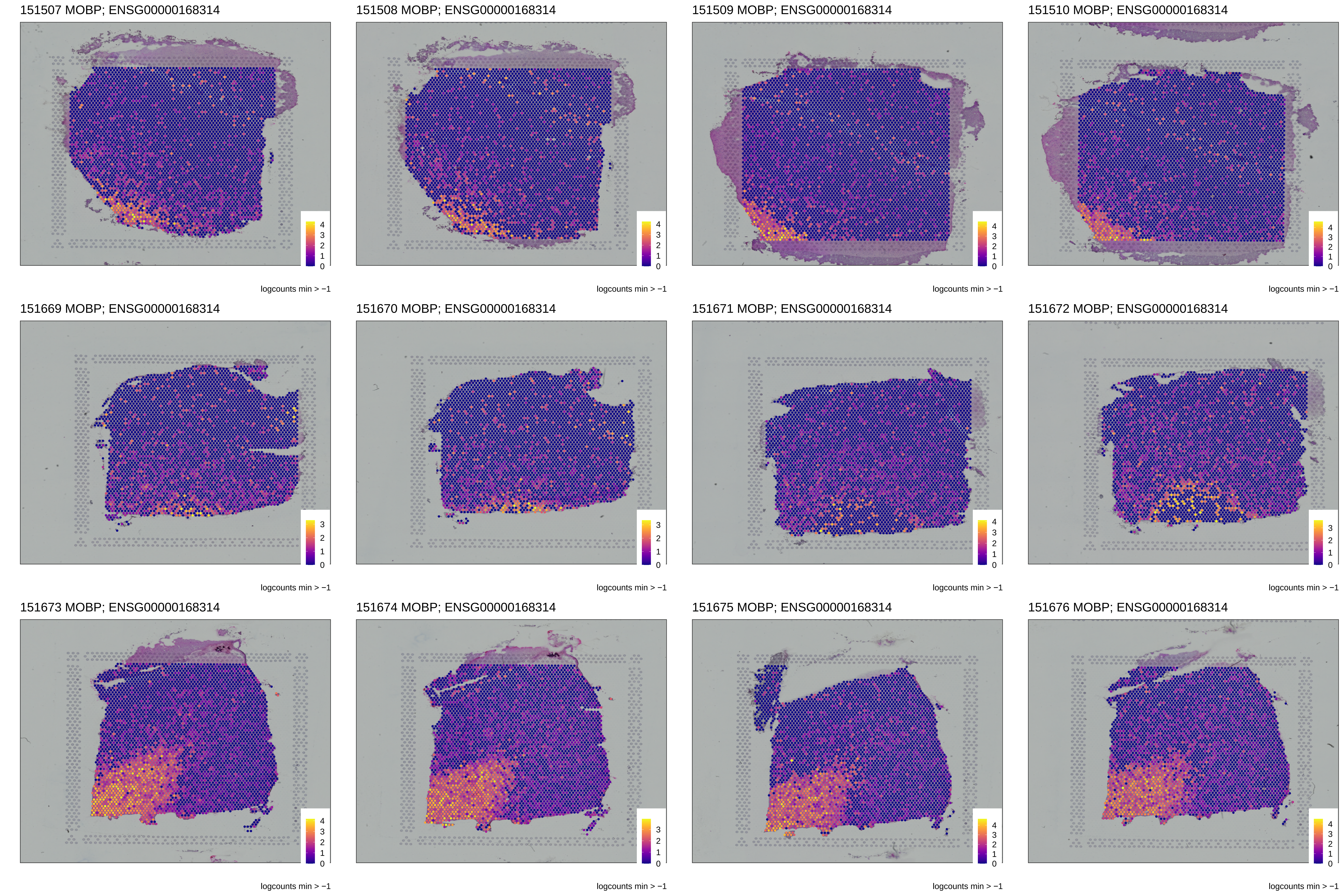
151507 MOBP; ENSG00000168314
4
3
2
1
0
logcounts min > −1
151508 MOBP; ENSG00000168314
4
3
2
1
0
logcounts min > −1
151509 MOBP; ENSG00000168314
4
3
2
1
0
logcounts min > −1
151510 MOBP; ENSG00000168314
4
3
2
1
0
logcounts min > −1
151669 MOBP; ENSG00000168314
3
2
1
0
logcounts min > −1
151670 MOBP; ENSG00000168314
3
2
1
0
logcounts min > −1
151671 MOBP; ENSG00000168314
4
3
2
1
0
logcounts min > −1
151672 MOBP; ENSG00000168314
3
2
1
0
logcounts min > −1
151673 MOBP; ENSG00000168314
4
3
2
1
0
logcounts min > −1
151674 MOBP; ENSG00000168314
3
2
1
0
logcounts min > −1
151675 MOBP; ENSG00000168314
4
3
2
1
0
logcounts min > −1
151676 MOBP; ENSG00000168314
4
3
2
1
0
logcounts min > −1
